# Food intake biomarkers for green leafy vegetables, bulb vegetables, and stem vegetables: a review

**DOI:** 10.1186/s12263-020-00667-z

**Published:** 2020-04-09

**Authors:** Elske M. Brouwer-Brolsma, Beate Brandl, Marion E. C. Buso, Thomas Skurk, Claudine Manach

**Affiliations:** 1 Division of Human Nutrition and Health, Wageningen University, PO Box 17, 6700 AA Wageningen, The Netherlands; 2 ZIEL Institute for Food and Health, Core Facility Human Studies, Technical University of Munich, Freising, Germany; 3 Else Kroener-Fresenius Center of Nutritional Medicine, Technical University of Munich, Freising, Germany; 4 Université Clermont Auvergne, INRA, UMR1019, Human Nutrition Unit, F63000 Clermont-Ferrand, France

**Keywords:** Lettuce, Spinach, Endive, Garden rocket, Asparagus, Artichoke, Celery

## Abstract

**Abstract:**

**Background:**

Numerous studies acknowledged the importance of an adequate vegetable consumption for human health. However, current methods to estimate vegetable intake are often prone to measurement errors due to self-reporting and/or insufficient detail. More objective intake biomarkers for vegetables, using biological specimens, are preferred. The only concentration biomarkers currently available are blood carotenoids and vitamin C, covering total fruit and vegetable intake. Identification of biomarkers for specific vegetables is needed for a better understanding of their relative importance for human health. Within the FoodBAll Project under the Joint Programming Initiative “A Healthy Diet for a Healthy Life”, an ambitious action was undertaken to identify candidate intake biomarkers for all major food groups consumed in Europe by systematically reviewing the existent literature. This study describes the review on candidate biomarkers of food intake (BFIs) for leafy, bulb, and stem vegetables, which was conducted within PubMed, Scopus and Web of Science for studies published through March 2019.

**Results:**

In total, 65 full-text articles were assessed for eligibility for leafy vegetables, and 6 full-text articles were screened for bulb and stem vegetables. Putative BFIs were identified for spinach, lettuce, endive, asparagus, artichoke, and celery, but not for rocket salad. However, after critical evaluation through a validation scheme developed by the FoodBAll consortium, none of the putative biomarkers appeared to be a promising BFI. The food chemistry data indicate that some candidate BFIs may be revealed by further studies.

**Conclusion:**

Future randomized controlled feeding studies combined with observational studies, applying a non-targeted metabolomics approach, are needed in order to identify valuable BFIs for the intake of leafy, bulb, and stem vegetables.

## Background

The importance of an adequate vegetable intake to promote health has been recognized for years. Vegetables are nutrient-dense foods containing high concentrations of dietary fibers as well as various vitamins, minerals, and phytochemicals. Accordingly, the potential impact of vegetable intake on human health has been extensively explored in nutritional epidemiological studies, showing beneficial associations [1–3]. Despite these suggested health effects, vegetable consumption rates among European citizens vary substantially, ranging from 34.4% of the population not consuming fruits and vegetables on a daily basis to 14.1% eating ≥ 5 portions [4]. Obviously, the wide variety of available vegetables substantially differs with respect to their nutrient content and associated benefits for health. Therefore, it is crucial to distinguish between the different vegetable groups, e.g., cruciferous vegetables, root vegetables, tubers, fruit vegetables, bulb and stem vegetables, and leafy vegetables, when addressing potential health effects.

Epidemiological data suggest that leafy vegetables deserve special attention. A large meta-analysis including 95 prospective studies recently revealed that a high intake of leafy green vegetables was associated with a reduced risk of coronary heart disease (10 studies, RR = 0.83, 95% CI = 0.75–0.91), stroke (4 studies, RR = 0.88, 95% CI = 0.81–0.95), and a 24% reduction in all-cause mortality for each 100 g/day increment in intake [1]. In another meta-analysis focusing on associations between all fruit and vegetable subtypes and type 2 diabetes, only green leafy vegetables (highest vs lowest intake categories) were associated with a significant risk reduction (RR = 0.84, 0.74–0.94) [2]. Spinach (*Spinacia oleracea,* Amaranthaceae family) represents a main member of leafy vegetables and has been shown to decrease postprandial arterial stiffness and systolic blood pressure in randomized controlled trials [5–7]. Moreover, garden rocket (*Eruca sativa*), a green-leafy vegetable from the Brassicaceae, has been proposed to possess anti-oxidative, anti-inflammatory, and anti-ulcer properties [8, 9]. Asteraceae is another botanical family of leafy vegetables representing amongst others endive and lettuce. Bulb and stem vegetables have been less extensively studied than leafy vegetables and constitute a rather heterogenous group including in particular celery, artichoke and asparagus. Celery (*Apium graveolens L.*) has been proposed to possess anti-oxidative and anti-inflammatory potency [10, 11], whereas artichoke (C*ynara scolymus*) received attention through its potential beneficial impact on LDL-cholesterol, endothelial NOS expression, and hepato-digestive function [12]. Asparagus (*Asparagus officinialis* with *Asparagales* as the botanical family) on its turn is a rich source of asparagine, potassium, vitamin C, and antioxidative glycolipids [13].

Up to now, vegetable intake in the epidemiological studies was generally assessed using traditional self-reported dietary assessment methods, such as food diaries, 24 h recalls and food frequency questionnaires (FFQs). Although these self-report dietary assessment methods provide valuable information, they are prone to measurement error and burdensome for both participants and researchers. Bingham and colleagues observed that the intake of fruits and vegetables assessed by FFQ was almost 2-fold higher compared with a food diary [14]. At the same time, the association between plasma vitamin C and the intake of fruits and vegetables was stronger when assessed by food diary than by FFQ, suggesting a higher degree of measurement error with the FFQ than with the food diary [14]. However, the processing of self-administered FFQs is much more convenient than the handling of diaries or recalls. To get insight in the habitual intake of commonly consumed foods, diaries and 24 h recalls need to be repeated at least 3 times [15] and even more frequently to capture specific types of vegetables and associated variety-specific nutrients [16]. Therefore, FFQs are usually the method of choice in large observational cohort studies. However, compared to diaries and 24 h recalls, FFQs are usually more restrained with respect to the variety of foods queried as well as portion size estimations. Moreover, both FFQs and recalls are prone to measurement error resulting from socially desirable answers and errors in food composition tables [17–20].

Therefore, the use of candidate biomarkers of food intake (BFIs), i.e., objective food consumption markers in biological specimens such as urine or blood, is gaining interest, even though there are still very few well-validated dietary intake biomarkers [21]. The intake of total fruit and vegetable consumption is frequently estimated using blood vitamin C or carotenoids (i.e., plasma α- and β-carotene, β-cryptoxanthin, and lutein) [22, 23]. However, the use of these biomarkers does not allow to distinguish between the types of fruit and vegetables consumed. Integrating the data as obtained by one or more of the traditional dietary assessment methods with validated BFIs data may eventually facilitate nutritional research by resulting in more precise intake estimates that have the ability to distinguish between the specific varieties of fruits and vegetables consumed [24].

To facilitate the identification and evaluation of new candidate BFIs, the Food Biomarker Alliance (FoodBAll) [21], a project funded by the Joint Programming Initiative a Healthy Diet for a Healthy Life, established guidelines to conduct a literature search dedicated to food intake biomarkers [25] and to evaluate their level of validation using a set of consensus criteria [26]. The guidelines were applied for all major food groups: fruit and vegetables, meats, fish, and other marine foods, dairy products, cereals and whole grains, alcoholic and non-alcoholic beverages, vegetable oils, nuts, and spices and herbs (http://foodmetabolome.org/wp3) [27–36]. The present article presents the results of the in depth exploration of possible biomarkers of intake for green leafy vegetables (lettuce, spinach, endive, and garden rocket) as well as bulb and stem vegetables (artichoke, asparagus, celery).

## Material and methods

### Selection of vegetable varieties

In order to cover the major varieties of vegetables consumed in Europe, FoodBAll aimed to cover cruciferous vegetables, root vegetables, tubers, leafy vegetables, fruit vegetables, and bulb and stem vegetables. In the present review, we focus on the most widely consumed leafy vegetables (lettuce, spinach, endive, garden rocket), and bulb and stem vegetables (asparagus, celery stalk, and artichoke). Herbs such as parsley, dill, basil, or peppermint and leafy cruciferous vegetables such as kale and cabbage are considered in another FoodBAll review in preparation.

### Literature search to identify putative BFIs

To identify relevant studies on leafy vegetables and bulb and stem vegetables, an extensive literature search was conducted following the Biomarker of Food Intake Reviews (BFIRev) methodology as proposed previously [25] built on the PRISMA statement (Preferred Reporting Items for Systematic reviews and Meta-Analyses). Briefly, a primary search was performed in the three databases Scopus, PubMed central, and Web of Science with the name of the specific vegetables and its botanical genus, i.e, (lettuce OR *lactuca sativa* OR escarole OR frisée), (spinach OR *spinacia oleracea*), (endive OR *cichorium endivia*), (garden rocket OR *eruca sativa* OR rucola OR rocket salad OR arugula OR rucoli OR rugula OR colewort roquette), (asparagus OR *asparagus officinalis*), (artichoke OR *cynara cardunculus* OR *cynara scolymus*), (celery OR *apium graveolens*) along with the common keywords: AND (urine OR plasma OR serum OR excretion OR blood) AND (human* OR men OR women OR patient* OR volunteer* OR participant*) AND (Biomarker* OR marker* OR metabolite* OR biokinetics OR biotransformation OR bioavailability OR ADME) AND (intake OR meal OR diet OR ingestion OR administration OR consumption OR eating OR drink*). Keywords were used in the fields [Topic], [All fields] and [Article Title/Abstract/Keywords] for Web of Science, PubMed, and Scopus, respectively. All searches were carried out in March 2017 and updated in March 2019. Only papers in English language were considered eligible; no restriction was applied with respect to the publication date. Human observational studies (cohort, case-control, cross-sectional studies) and human intervention studies (randomized controlled trials, acute, short-term, or long-term studies) were considered eligible. After duplicates removal, a first selection of papers was performed according to abstract and title relevance, thereafter full-text screening was performed.

### Evaluation of the identified candidate biomarkers

The quality of the individual candidate BFIs was assessed by evaluating their plausibility and specificity, dose-response, time-response, robustness, reliability, stability, performance, and reproducibility of their analysis method [26]. The name of the potential biomarkers and their synonyms were queried in the previously mentioned databases in combination with AND (biomarker* OR marker* OR metabolite* OR biokinetics OR biotransformation OR pharmacokinetics OR bioavailability OR ADME) to collect the relevant information for each identified candidate biomarker. In addition, the databases HMDB (https://www.hmdb.ca), FooDB (http://foodb.ca/), Phenol-Explorer (http://phenol-explorer.eu/), Dictionary of Food Compounds (http://dfc.chemnetbase.com/faces/chemical/ChemicalSearch.xhtml), Duke’s phytochemical and ethnobotanical databases (https://phytochem.nal.usda.gov/phytochem/search), eBASIS, (http://ebasis.eurofir.org/Default.asp), Knapsack (http://kanaya.naist.jp/knapsack_jsp/top.html) and PhytoHub (http://phytohub.eu) were searched to identify all possible sources of each candidate biomarker and thus check its specificity. The validation level of the candidate BFIs was evaluated via the pre-defined criteria set by Dragsted and colleagues [26], which were based on eight questions related to the biological and analytical aspects: Q1: is the marker compound plausible as a specific BFI for the food or food group (chemical/biological plausibility)? Q2: is there a dose-response relationship at relevant intake levels of the targeted food (quantitative aspect)? Q3: is the biomarker kinetics described adequately to make a wise choice of sample type, frequency, and time window (time-response)? Q4: has the marker been shown to be robust after intake of complex meals reflecting dietary habits of the targeted population (robustness)? Q5: has the marker been shown to compare well with other markers or questionnaire data for the same food/food group (reliability)? Q6: is the marker chemically and biologically stable during bio specimen collection and storage, making measurements reliable and feasible? Q7: are analytical variability (CV%), accuracy, sensitivity, and specificity known as adequate for at least one reported analytical method? Q8: has the analysis been successfully reproduced in another laboratory (reproducibility)? This set of criteria therefore reflects the current level of validation of that particular compound and pinpoints the additional research needed to increase its validation.

## Results and discussion

### Leafy vegetables

**Fig. 1:**
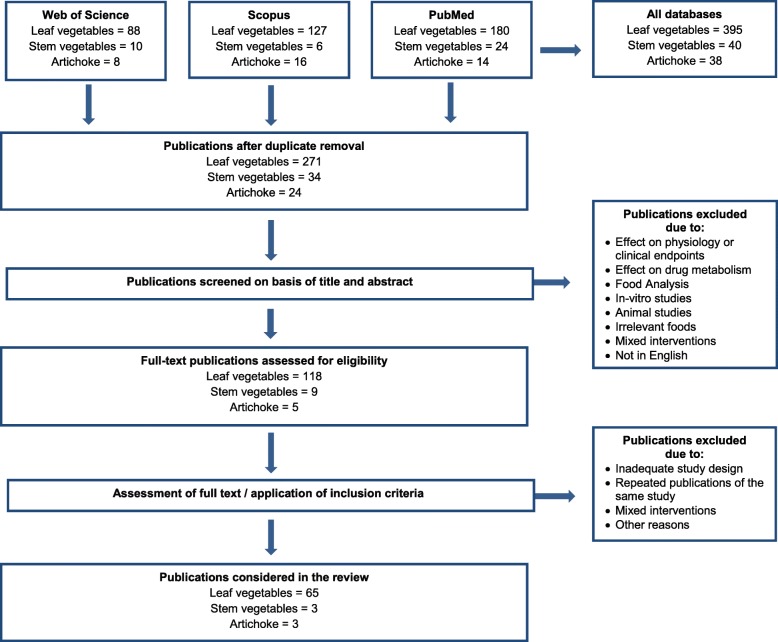
Flow diagram of study selection

The primary literature search on leafy vegetables resulted in 361 articles. After removal of duplicates, 247 publications were screened based on their title and abstract, 108 publications underwent full-text assessment, and eventually 61 publications were included in this review. In March 2019, the primary literature search was updated. The three databases yielded 34 new hits of which 24 remained after removal of duplicates, ten publications remained after title/abstract screening and four were selected based on the full-text assessment (Fig. 1). Main reasons for exclusion were that the: (1) intake assessment was too general (i.e., vegetable intake), (2) focus was on the effect of the intake on health-related markers, and/or (3) exposure in a nutritional intervention study was not only green leafy vegetables. Finally, the two searches combined resulted in 65 potentially relevant papers that provided data on the intake of one or more of the selected leafy vegetables (lettuce *n* = 7, endive *n* = 1, rocket salad *n* = 1, spinach *n* = 65) and measured one or more related compounds in body tissue, such as plasma, urine, and/or feces. The main characteristics of these studies are displayed in Additional file 1: Table S1.

A wide range of metabolites were reported in biofluids after consumption of leafy vegetables. However, as discussed below, many of these metabolites have other dietary origins and were therefore not specific enough to be retained as candidate BFIs. Carotenoids and their derivatives and conjugates were the most commonly reported metabolites after consumption of leafy vegetables. Carotenoids belong to the family of tetraterpenoids; where oxygen-containing carotenoids are called xanthophylls (e.g., lutein and zeaxanthin) and unoxygenated carotenoids are called carotenes (e.g., α- and β-carotene, and lycopene). As carotenoids are commonly found in a wide range of vegetables, they were considered unspecific and as such not retained as candidate BFIs. To illustrate, a comparative study across five European countries showed that spinach was the food contributing most to the total lutein intake in France, Spain, and the Netherlands (i.e., 30–34%) [37]. Lettuce was the second major food contributing to lutein intake in France and Spain (i.e., 8–16%) [37]. However, lutein is also present in egg yolk, pistachio, red pepper, pea, broccoli, corn, and common herbs [38, 39]. Additionally, spinach has been shown to be a major food contributing to the total β-carotene intake (12-26%). Though α- and β-carotene are primarily obtained through the consumption of carrots [37]. Besides the studies on carotenoids, two observational studies reported that vitamin K1 (phylloquinone) was associated with the consumption of spinach and lettuce [40, 41]. However, phylloquinone is widely distributed in all photosynthetic plants, where it serves as a cofactor for photosynthesis and is also available as dietary supplement [42]. Moreover, neither vitamins nor nitrate or phosphate were retained as candidate biomarkers for BFIs as these components are common in a range of other foods as well [43, 44]. Finally, some intervention studies reported an increased urinary oxalate excretion after consumption of spinach. Indeed, it is well-known that spinach should be restricted by individuals at high risk of calcium oxalate kidney stones [45]. However, beetroots, rhubarb, chocolate, wheat bran, almonds, and peanuts are other important sources of oxalic acid, making it not specific enough to represent a promising candidate biomarker of intake for spinach [45].

After excluding articles reporting on obviously non-specific biomarkers, six articles provided data on candidate BFIs of green leafy vegetables (Table 1). The retained articles included five acute single-dose studies and one cross-over intervention focusing on either urinary and/or blood-based biomarkers (i.e., plasma). Sample sizes were small, ranging from 3 up to 11 participants. Five studies used a targeted approach; one study applied untargeted metabolomics. In total, 9 candidate BFIs were identified for spinach, 2 for lettuce, and 2 for endive. None of the selected articles reported on candidate biomarkers for rocket salad.

**Table 1:** Overview of the selected studies on green-leafy vegetables

Dietary factor	Intervention	Dose of intervention	Study design	*n*	Analytical method	Sample type	Candidate biomarkers of food intake	Primary reference
Spinach								
	Just-boiled fresh spinach	1.2 kg	Acute single dose	8	HPLC-UV	Plasma (after 3 h)	Chlorophyll-related compounds (CRCs) (pheophytin (Phe) and pheophorbide (Pho))	[46]
	Vegetable together with fried rice	200 g	First study: acute single doses (4 distinct treatments: spinach, celery, onion, no vegetables) Second/third studies confirmation: singles doses or combinations	10 (and 3 for secondand third)	LC−MS (UPLC and Q-TOF MS) (untargeted metabolomics)	Urine (up to 7 h)	4-guanidino-butanoic acid with an Isoprene modification (tentative identification)	[47]
	Spinach-containing diet–frozen spinach thawed and cooked	453 g spinach/day	2-arm cross-over intervention (2 × 3 weeks)	7	Atomic absorption spectrophotometry (Faecal, urinary, and dietary calcium and zinc levels) kits for oxalate and hydroxyproline-assays	Urine	Hydroxyproline	[48]
	Freeze-dried ^13^C-labeled spinach	5 g (providing 160 μmol methoxyflavonols, including 70 μmol TMM4’-glucuronide)	Acute single dose	5	UHPLC-MS^n^	Plasma (up to 24 h)	^13^C_17_TMM-glucuronide ^13^C_17_TMM-sulfate ^13^C_16_Patuletin-glucuronidesulfate-methyl ^12^C_1_^13^C_16_Spinacetinglucuronide-sulfate ^13^C_17_Spinacetinglucuronide-sulfate	[49]
Lettuce								
	Fresh lettuce	250 g	Acute single dose	11	HPLC-Coularray	Plasma (up to 6 h)	ρ-coumaric acid Caffeic acid	[50]
Endive								
	Endive soup (300 g) with a slice of white bread	150 g (precooked mass) of endive = 9 mg kaempferol	Acute single dose	8	LC/MS	plasma and urine (up to 24 h)	Kaempferol-3-glucoside Kaempferol-3-glucuronide	[51]

### Spinach

Spinach has been considered since ancient times as a food with functional properties that supports health maintenance [52, 53]. It is of high nutritional value, with large contents of folates, vitamin A, C, and K, and minerals such as iron, magnesium, and manganese, allowing a substantial contribution to their respective recommended dietary allowances when consuming a single serving [53]. Spinach also provides nitrates that—after conversion by salivary bacteria and host reductases into nitrites—improve the nitric oxide status and as such has been beneficially related to vascular function and blood pressure [6, 7]. Spinach also contains a number of phytochemicals, including chlorophylls, carotenoids (lutein, neoxanthin, zeaxanthin), and chloroplast-specific lipids, e.g., mono- and di-galactosyldiacylglycerol (MGDG and DGDG, respectively) and the anionic sulfolipid, sulfoquinovosediacylglycerol (SQDG). It also provides unique flavonoids, e.g., derivatives of patuletin, spinacetin, spinatoside, jaceidin, and methylenedioxyflavone [54–56].

Of the four identified studies that examined changes in metabolites associated with spinach intake, none had the objective to identify BFI (Table 1). Although it is generally assumed that chlorophyll derivatives have very low bioavailability [57], Chao and colleagues (2018) reported on the identification of chlorophyll-related compounds, i.e., pheophytin (FDB007170) and pheophorbide (FDB031104) derivatives, in plasma 3 h following a high acute dose of just-boiled fresh spinach [46]. Pheophytin and pheophorbide are degradation products of chlorophylls that lack the central Mg^2+^ ion and are readily produced during thermal processing or acidification [57]. It is commonly known that chlorophylls and derivatives are ubiquitous in all photosynthetic plants with particularly high levels in leafy green vegetables. Chlorophylls and derivatives are thus unlikely to be sufficiently specific for spinach, even when used in combination with other spinach derived compounds [58].

A pilot metabolomic study explored the changes in the urine metabolomes of 10 participants after an acute intake of 200 g spinach in the context of a controlled diet, compared to the intake of 200 g celery, 200 g onion, or to the controlled diet without addition of any vegetable [47]. The principal component analysis score plot showed a shift in the post-spinach urine metabolome compared to the control group and intake of other vegetables. However, among the signals discriminating spinach intake, the authors only reported one discriminating ion with m/z 214.15 that was tentatively identified as 4-guanidino-butanoic acid with an isoprene modification. When the identification is confirmed, the validation criteria proposed by the FoodBAll consortium BFI will have to be assessed for this compound. Similar intervention studies using untargeted metabolomics with large analytical coverage will be of great value to identify other candidate biomarkers of spinach intake, provided that the discriminating signals can be properly identified.

Another selected study was a cross-over intervention with 3 weeks supplementation that aimed at comparing the effects of spinach and cheese on the calcium and zinc balance, oxalate excretion, and urinary hydroxyproline in 7 post-menopausal women [48]. An increased urinary excretion of hydroxyproline (PubChem CID: 5810) was reported. However, this compound is a common amino acid and a constituent of collagen that has also been suggested as a biomarker for processed meat consumption and can therefore be excluded as candidate biomarker for spinach [59].

The last relevant study aimed at identifying the polyphenol metabolites recovered in human plasma after ingestion of 5 g freeze-dried ^13^C-labeled spinach. The main metabolites were a glucuronide and a sulfate of 5,3′,4′-trihydroxy-3-methoxy-6,7-methylendioxyflavone (TMM), and spinacetin-glucuronide-sulfate [49]. The secondary search in the literature and in various databases (FooDB, PhytoHub, Duke’s phytochemical and ethnobotanical database, Phenol-Explorer, CRC press Dictionary of Food Compounds) revealed that the derivatives of spinacetin and TMM are compounds with a high specificity for spinach. As specificity is an important prerequisite for BFIs, the metabolites of spinacetin and TMM identified in plasma deserve further investigation as putative intake biomarkers, with the examination of the other validation criteria. Such derivatives of the spinach flavonoids are likely to be also present in urine samples. However, they were not targeted in the analyses conducted on urine samples so far.

Thus, the available literature is scarce on putative BFIs for spinach. However, a few studies as well as the specific phytochemical composition of spinach suggest that further studies designed for identification of BFIs and using an exploratory metabolomic approach should reveal specific candidate biomarkers for this leafy vegetable. As spinach can be consumed either in raw (salads, smoothies) or cooked forms (steamed, boiled, pan-fried, in soups), it will be of interest to assess the performance of the candidate BFIs for the various forms consumed.

### Lettuce and endive

Lettuce and endive (Asteraceae family) are leafy vegetables with a rich nutrient profile that is rather comparable to spinach (Amaranthaceae family). The presence of sesquiterpene lactones in lettuce and endive is what sets the two species apart, which is immediately noticed when consuming the vegetables owing to their bitter taste [60, 61]. Lactucin, lactucopicrin, 8-deoxylactucopicrin, lettucenin A, and lactuside A are the main sesquiterpene lactones reported in lettuce and endive [61]. However, the presence of these compounds and their metabolites in human biofluids and their possible usefulness as biomarkers of intake have not been considered so far.

We identified only one article that examined metabolite changes after lettuce intake (Table 1), which detected ρ-coumaric acid (PubChem CID: 637542) and caffeic acid (PubChem CID: 689043) in plasma 6 h following an acute single dose of lettuce [50]. However, ρ-coumaric acid is also present in many other foods, ranging from fruits (e.g., apples, oranges, grapes, berries), vegetables (e.g., beans, potatoes, onions) to cereals (e.g., maize, oats, and wheat) [62]. As reported based on the Bavarian Food Consumption Survey (Germany), caffeic acid is mainly obtained through the intake of coffee (92%) followed by fruit consumption [63]. Both caffeic and p-coumaric acid have a too broad distribution in food to be used as BFIs, even in combination with other compounds. The only potentially relevant article for endive reported on the identification of kaempferol-3-glucoside (PubChem CID: 5282102) and kaempferol-3-glucuronide (PubChem CID: 22846027) in urine and plasma following a single dose of 300 g thick endive soup consumed with a slice of white bread and a glass of water (Table 1) [51]. Once more, these compounds are also present in a wide variety of other plants [64]. According to Phenol-Explorer, kaempferol and its glucoside have been reported in high content in many herbs and spices, in tea, common beans, in kale and other cruciferous vegetables, as well as in Swiss chard leaves and in spinach.

Thus, eventually, none of the compounds resulting from the systematic search for green leafy vegetables were sufficiently promising to be evaluated on the validation criteria. Yet, metabolites of sesquiterpene lactones may deserve further study as putative BFIs.

### Bulb and stem vegetables

The literature search in the three databases resulted in 40 papers considering asparagus or celery and 38 papers considering artichoke. Most papers reported on health effects rather than on candidate biomarkers of intake, used in vitro and animal models, and particularly focused on immune-modulation upon different asparagus-preparations. As such, these studies did not match our inclusion criteria and were excluded. After removal of duplicates, title/abstract screening, and full-text screening, eventually six papers were considered relevant for this review (asparagus (*n* = 2), artichoke (*n* = 3), and celery (*n* = 1)) (Table 2).

**Table 2:** Overview of the selected studies on bulb-and stem vegetables

Dietary factor	Intervention	Dose of intervention	Study design	n	Analytical method	Sample type	Candidate biomarkers of food intake	Primary reference
Asparagus	Freshly boiled Asparagus	100 g	Acute intervention (single dose intervention)	8	GC-MS	Urine	S-methyl-thioacrylate S-methyl-3- (methylthio)-thiopropionate Dimethyl trisulfide Tetrahydrothiophene	[65]
	Asparagus	500 g	Acute intervention (single dose intervention) Repetition three months later	8 3	GC-MS	Urine (overnight collection after asparagus dinner)	Methanethiol, Dimethyl sulphide Dimethyl disulphide Bis-(methylthio)methane Dimethyl sulphoxide Dimethyl sulphone	[66]
Artichoke	Steam cooked artichoke	61.7 g with 5.5 g olive oil	Acute intervention (single dose intervention)	5	HPLC-Coularray	Plasma (7 consecutive samples during 8 h)	Hydroxycinnamic acid Dicaffeoylquinic acid	[67]
	Artichoke capsules	320 mg extracts	Acute intervention (3 doses during 8 hours, 0, 4 and 8 hours)	10	HPLC	Urine (24 h urine vs. baseline)	Ferulic acid Isoferulic acid Dihydroferulic acid Vanilic acid	[68]
	Artichoke leaf extract (ALE)	2 different high-dose artichoke leaf extracts – (ALE): ALE A 2.4 g and ALE B 0.625 g	Acute intervention (2 different doses, cross-over, separated by 10 days wash out	14	HPLC	Blood and urine (13 blood samples over 12 h, 1 urine sample after 24 h)	Caffeoylquninic acid Dihydroferulic acid Ferulic acid Dihydrocaffeic acid	[69]
Celery	Celery leaves	2 g/kg body weight	Controlled acute Study (single dose intervention)	20	HPLC	Plasma (9 samples over 28 h	Apigenin	[70]

### Asparagus

Asparagus spears (*Asparagus officinalis* with *Asparagales* as the botanical family) are frequently consumed in various areas throughout Europe and asparagus preparations have been considered to exhibit distinct health effects [13, 71]. Due to these suggested health effects, the refined taste of Asparagus, and the odorant urine associated with its consumption, the ingredients of Asparagus are matter of intense investigation [72]. More than 100 of volatile compounds have been detected in asparagus, as well as rutin, a glycoside of the widely distributed quercetin, and glutathione in high content [73]. Asparagus is a rich source of asparagine, potassium, and vitamin C. Two compounds seem specific for asparagus, specifically asparagusic acid [73, 74], a heterocyclic compound with two sulphur atoms, and the saponin protodioscin that is only present in fenugreek in the human diet. Documentation of potential biomarkers is sparse. From the literature screening only two papers were retained for this review.

White and colleagues used GC-MS to identify the odor-bearing compounds in urine samples of 8 participants, after the intake of 100 g asparagus freshly boiled for 10 min, and reported on the identification of S-methyl-thioacrylate (also known as 2-propenethioic acid, PubChem CID: 543202), S-methyl-3-(methylthio)-thiopropionate, tetrahydrothiophene, and dimethyl trisulfide [65]. Moreover, Waring and colleagues provided 500 g asparagus spears to 8 participants and identified six sulphur-containing alkyl compounds identified as methanethiol, dimethyl sulphide, dimethyl disulphide, bis-(methylthio)methane, dimethyl sulphoxide, and dimethyl sulphone in overnight urine samples following asparagus dinner [66]. Consumption of asparagus is known from ancient times to cause odorous urine, often described as rotten cabbage-like. However, the inter-individual variation for the production of odorous urinary metabolites after asparagus intake has been intriguing for centuries and is not yet fully elucidated. We now know that apart from differences in the production of odorous metabolites, some individuals are unable to smell the asparagus-associated odor in urine, due to a specific anosmia related to genetic polymorphisms near the olfactory receptor gene OR2M7. In a well-controlled study taking into account anosmia, 92% of the 37 volunteers produced odorous urine, although with differences in intensity [75].

In conclusion, while odorous urine is frequently recognizable after asparagus consumption likely due to highly volatile metabolites of asparagusic acid [66], these metabolites may not represent appropriate quantitative BFIs because of the inter-individual variation in their production. So far, all research was performed with GC-MS on urine samples. Other non-volatile metabolites specific for asparagus may be identified with LC-MS, possibly in plasma as well.

### Celery stalks

Celery stalks (*Apium graveolens L.*) from the family of Apiaceae / Umbelliferae have a Mediterranean origin and are often used for flavoring as food or as extract. Their biological effects have been little studied so far [10]. Regarding celery, seven publications were screened on basis of title and abstract, of which one paper reported on a potential candidate BFI for celery, namely, apigenin [70]. Apigenin belong to the flavone family and its content is reported to be 1.3–10.8 mg/100 g fresh celery (*Apium graveolens var. dulce*) [76]. However, apigenin is not unique to celery. It is also present in high amount in parsley and Chamomile, and distributed in many other foods in lower concentrations [76]. There is still too little information about the bioavailability of apigenin after consumption of its different sources, its metabolism, pharmaco-kinetics, and dose-response, to state whether apigenin might be useful as a component of a combined BFI for celery.

### Artichoke

Artichoke (C*ynara scolymus*), from the family of Asteraceae, has been used for centuries as food and herbal medicine, already by the ancient Egyptians, Greeks, and Romans who considered it as a digestive aid and a treatment for hepato-digestive diseases [77]. It has more recently received substantial attention for its potential beneficial effects on dyslipidemia, e.g., lowering blood LDL-cholesterol and triglycerides [78], stimulation of bile secretion, prebiotic effects, upregulation of endothelial NOS expression [12], and for its hepato-protective effects [79]. These health benefits have been attributed to the synergy between inulin, a soluble fiber of the fructans family and polyphenols such as cynarin (1,3-O-dicaffeoylquinic acid; HMDB0029279), chlorogenic acid, and luteolin glycosides [77, 79]. Artichoke leaves extracts are also consumed as herbal supplement and many of the published studies were conducted using such supplements. Of the 38 retrieved publications for artichoke, three were included in this review. All of these were acute intervention studies with 5–14 volunteers. Whereas Azzini et al. used cooked vegetable (61.7 g administered in a single dose) [67], Rechner used capsules with 320 mg extracts in three doses during 8 h [68] and Wittemer used high-dose leaf extracts with standardized flavonoid content [69] (Table 2). These publications reported on hydroxycinnamic acid [67], dicaffeoylquinic acid [68], caffeoylquinic acid, and dihydroferulic acid [69], which were either measured in plasma (Azzini), urine (Rechner), or both (Wittemer). As these components are identified after consumption of many other food items as well (e.g., plum, coffee beverages), none of these compounds can therefore be followed up a specific BFI, even for used in combined BFI.

### The way forward

Based on the identified studies in this literature review it is clear that there is a need for new intervention studies with well-controlled experimental diets to successfully identify potential BFIs for lettuce, spinach, endive, garden rocket, asparagus, celery, and artichoke. In particular, studies applying untargeted analyses with large analytical coverage are warranted. All the studies identified in this review, except one, used a targeted approach. Targeted analyses have a great selectivity and sensitivity, but only assess a limited number of known metabolites. Untargeted analyses measure hundreds of metabolites at once in an exploratory manner and therefore provide a more effective route to detect potential interesting new candidate BFIs when our knowledge is too limited. A combination of two or more of the metabolites identified with untargeted analyses, instead of a single biomarker is a promising approach to establish more specific and robust BFIs for lettuce, spinach, endive, garden rocket, asparagus, celery, and artichoke. Observational studies with precise data on dietary intake as well as biological samples are needed as a second step to assess the specificity and robustness of the candidate BFIs. Possible confounding factors can only be identified in large free-living populations eating complex non-controlled diets and it is preferable to assess the applicability of the candidate BFIs in several populations with different dietary background [80–82].

## Conclusions

The extensive literature search conducted under the well-defined criteria of the BFIRev protocol demonstrated that the field has only been poorly studied so far and that there are no specific candidates BFIs for leafy vegetables yet. However, the food chemistry data indicate that some fairly specific compounds exist for the vegetables under study. Future well-designed studies, including randomized controlled feeding studies as well as observational studies, applying an untargeted metabolomics approach, may thus yield new candidate BFIs for the intake of green leafy vegetables.

## Supplementary information

**Additional file 1:.** Table S1. Overview of all potentially relevant studies on green leafy vegetables [6, 40, 41, 46–49, 51, 83–144]

## Data Availability

Not applicable.
