# Supplementary material for: Food intake biomarkers for green leafy vegetables, bulb vegetables, and stem vegetables: a review
Source: Genes Nutr. 2020 Apr 9;15:7. doi: 10.1186/s12263-020-00667-z (PMC7144047; doi:10.1186/s12263-020-00667-z)
Supplement: Supplementary file 1 — Additional file 1:. Table S1. Overview of all potentially relevant studies on green leafy vegetables [6, 40, 41, 46–49, 51, 83–144] [file 12263_2020_667_MOESM1_ESM.docx]

Table S1. Overview of all potentially relevant studies on green leafy vegetables

| **Dietary**  **factor** | **Intervention** | **Dose of intervention** | **Study design** | ***n*** | **Analytical method*** | **Sample type** | **Candidate Biomarkers of Food Intake** | **Primary Reference** |
| --- | --- | --- | --- | --- | --- | --- | --- | --- |
| **Spinach** |  |  |  |  |  |  |  |  |
|  | Spinach-carrot meal  (intervention 1) and  green-leafy vegetables (GLV) (intervention 2) | 7.9 mg β-carotene and 130 mg ascorbic acid (meal)  100 g GLV/d alone | (1) Acute single dose  (2) Parallel 5-arm intervention study (3 weeks) | 8  40 | Spectrophotometry and atomic absorption spectrometry | plasma (over 4h) | **β-carotene**  **zinc**  **vitamin C** | (84) |
|  | Fresh-frozen spinach added to high-fat hospital diet | 100 g/d | Single arm intervention study (3-days) | 54 | Enzymatic colorimetric technique (oxalate)  Hydrolysis and petroleum ether extraction (Faeces fat) | urine  faeces | **oxalate (urine + faeces)** | (85) |
|  | Boiled and stir fried spinach (oil + salt) | 200 g (fresh weight)/d - containing 8·6mg b-carotene, 13·4 mg lutein, 6·5mg violaxanthin and 3·0mg neoxanthin | Single arm intervention study (1-week wash out period followed by 1 week spinach) | 5 | HPLC-MS/ high resolution MS  NMR spectra | plasma | **β-carotene**  **lutein** | (86) |
|  | 1,236 mg oxalate from spinach intake (no indication fresh amount/cooking) | 1,236 mg oxalate from spinach intake (no indication fresh amount/cooking) | Acute single dose | 8 | GC + enzymatic colorimetric technique | urine (over 8h) | **oxalate** | (87) |
|  | Grilled spinach (from frozen spinach) (alone or with 100g dairy product + 20g oil) | 100 g | Acute single dose | 10 | HPLC-UV/Vis | urine (over 6h and 24h) | **oxalate** | (88) |
|  | Liquid spinach powder preparation | 10 g/d | Single-arm crossover intervention study (8-weeks –3 food products from which one was spinach over 2 weeks) | 23 | Titration  DCA method  Spectrometry  Test kits  Microtiter plate assay  FRAP-assay  Gas chromatography | plasma, plasma lipoprotein (LPL) subfractions | **β-Carotene**  ***cis*-β-Carotene**  **α-Carotene**  **β-Cryptoxanthin**  **lutein (incl. zeaxanthin)**  **all-*trans*-Lycopene**  ***cis*-Lycopene**  **phytofluene**  **phytoene** | (89) |
|  | Cooked chopped spinach | 260 g | Acute crossover single doses (3 doses of 3 different products (one spinach) separated by 2 –weeks) | 16 | HPLC-UV/Vis  enzymatic colorimetric method (triacylglycerols) | chylomicrons fraction of plasma (up to 9h) | **lutein**  **triacylglycerols** | (90) |
|  | Frozen spinach products:  - Whole leaf spinach  - Minced spinach  - Liquefied spinach  - Liquefied spinach + added dietary fibre (10g/kg wet weight) | 20 g/MJ/d | Parallel 6-arm intervention (3-weeks of 4 spinach products or  β-carotene supplement or control) | 70 | HPLC-DAD  Kits for enzyme activity assay | serum | **α-tocopherol** | (91) |
|  | Frozen spinach products:  - Whole-lead spinach  - Minced spinach  - Liquefied spinach  - Liquefied spinach + added dietary fibre (10g/kg wet weight) | 20 g/MJ/d | Parallel 6-arm intervention (3-weeks of 4 spinach products or  β-carotene supplement or control) | 70 | Spectrophotometry  HPLC-UV | serum | ***all-trans*-β-carotene**  ***cis-*β-carotene (β-carotene)**  **lutein**  **α-carotene**  **retinol**  **lycopene** | (92) |
|  | Frozen spinach products:  - Whole-lead spinach  - Minced spinach  - Liquefied spinach  - Liquefied spinach + added dietary fibre (10g/kg wet weight) | 20 g/MJ/d | Parallel 6-arm intervention (3-weeks of 4 spinach products or  β-carotene supplement or control) | 70 | Enzyme-immunoassay | plasma | **folate** | (93) |
|  | Spinach breakfast drink | 294 g | Acute single dose cross-over (one spinach among 5 interventions during a period of 10-week) | 8 | Spectrophotometry  HPLC-UV  Oxygen radical absorbance capacity (ORAC) assay, Trolox equivalent antioxidant capacity (TEAC) assay and ferric reducing ability (FRAP) assay | serum (0-4h/11h) | **Urate** | (94) |
|  | Boiled fresh spinach | 1.2 kg | Acute single dose | 8 | HPLC-UV/Vis | plasma (after 3h) | **Chlorophyll-related compounds (CRCs)** (**pheophytin (Phe) and pheophorbide (Pho))**  **lutein**  **zeaxathin** | (52) |
|  | Spinach frittata (egg white + frozen, chopped spinach) | Dose that provides 6mg lutein/d | 4-arm cross-over intervention study (4 treatments from which one spinach) (9d) | 10 | HPLC-PDA | serum and TRF | **lutein** | (95) |
|  | Spinach-based meals (microwave-heated spinach in a spinach-based meal with or without Ca supplementation) | 270 g (8.61 mg carotenoids/100 g fresh weight) | Acute cross-over single doses (3 interventions with 1-week washout periods) | 25 | HPLC-coupled UV/Vis-PAD | plasma (up to 10h) | **Lutein**  **β-Carotene**  **β-Cryptoxanthin** | (96) |
|  | Cooked and uncooked spinach | Food daily intake assessed by interview (previous 12 months estimation) | Cross-sectional analysis | 61 | Inductively-coupled plasma mass spectrometry (ICP/MS) | whole blood | **lead** | (97) |
|  | ^15^N-labeled spinach soup | 308 μg labeled total flavins (Free riboflavin + flavin mononucleotide (FMN)) as ^15^N-labeled spinach soup with simultaneous 200 μg^13^C-labeled riboflavin | 2-arm crossover intervention (single doses on 2 occasions 4 weeks apart: milk or spinach) | 20 | Stable-isotope labelling method  HPLC-ESI-MS | plasma (over 7 hours)  urine (over 24h) | **riboflavin**  **riboflavin** | (98) |
|  | Mean spinach intake | Food record | Observational study | 46 | No information in abstract | plasma (end of each diet record) | **vitamin K_1_ (phylloquinone)** | (40)  Only abstract available. |
|  | Spinach | 175g spinach (1,570 mg/d oxalate content) | 2-arm crossover intervention (4 weeks for spinach among other products) | 8 | Atomic absorption spectrophotometry (calcium) and urine kit for oxalate + faeces (after drying/dilution/heating) | urine and faeces  (all during last 14d) | **oxalate (urine + faeces)** | (99) |
|  | Raw trimmed spinach + either 20g or 1g fat and 6.0 μmol [2H4] retinyl acetate (d_4_-RA) | Spinach containing 6 mg of β-carotene | Acute crossover single doses (4 test meals 2-6 weeks apart spinach or carrot | 3 | HPLC-MS | plasma (LPL-rich fraction) (over 8.5 hours) | ***all-trans*-β-carotene**  **α-carotene**  **lutein**  **RE-derived retinol**  (response when fat added) | (100) |
|  | Chopped or whole-leaf cooked spinach  (oil + yoghurt) | Spinach meal with 15mg lutein and 10mg β-carotene | Acute crossover single dose | 7 | HPLC-UV/Vis | ileal effluent (up to 24h) | **β-carotene**  **lutein** | (101) |
|  | Spinach juice | 375 mg of oxalate | Acute single dose (12 months after bariatric surgery) | 91 | Enzymatic and colorimetric methods | urine (after 6 hours)  serum | **oxalate**  **citrate** | (102) |
|  | Trimmed fresh spinach | 50 g or 150 g | Acute single doses crossover intervention | 11 | HPLC-fluorometric detector | serum (up to 9h) | **vitamin K_1_ (phylloquinone)** | (103) |
|  | Boiled spinach with (25g) butter or without | 227 g boiled spinach | Acute single doses (3 interventions over 3 weeks: control + 2 spinach) | 5 | HPLC-UV | serum (over 10h and after 24h) | **vitamin K (phylloquinone)** | (41) |
|  | Test meal cooked fresh spinach + bacon + bread and beer | No information | Acute single dose | 23 | Chemiluminescence + MS | whole blood (over 24h) | **nitrosamines**  ***(N*-Nitrosodimethylamine**  ***N*-Nitrosodiethylamine)** | (104) |
|  | Dried spinach powder | 10.4g/d (15 mg of lutein and zeaxanthin/95% lutein + 2 mg β-carotene) | Parallel 2-arm intervention studie (8 weeks – carrot or spinach) | 52 | HPLC-DAD | serum | **lutein**  **zeamanthin** | (105) |
|  | Spinach (frozen) with or without corn | Spinach (60 g/day) with 150g corn for 10 subjects | Parallel 3-arm intervention (15-weeks; spinach spinach+corn or corn only) | 11 | HPLC-PAD | serum (at 4 to 15 weeks) | **lutein**  zeaxanthin (NP)  β-carotene (NP) | (106) |
|  | Canned spinach | 25 g spinach containing 120mg oxalic acid | Acute single doses crossover (sugar beet, spinach or sodium oxalate (control)) | 9 | Enzymatic titration method | urine (over 24h) | **oxalate** | (107) |
|  | Cooked, pureed and sauteed Indian spinach (Basella alba) | 750 μg/d retinol equivalent (RE) | 3-arm parallel intervention study (60 days; sweet potato, or spinach or control) | 14 | Deuterated-retinol (stable isotope)-dilution technique | plasma | **vitamin A**  **β-carotene** | (108) |
|  | Boiled spinach | 280 g/d providing 12,700 μg carotene | 2-arm intervention (2 weeks; either carrot or spinach) | 17 | No information | plasma | **retinol**  **carotene** | (109)  Only abstract available. |
|  | Spinach with corn frozen product | Spinach (60 g/d) and corn (150 g/d) | Single-arm 15-wk intervention (spinach + corn) | 7 | HPLC-DAD | serum  buccal mucosa cells | **lutein**  **zeaxanthin**  l**utein**  **lutein** | (110) |
|  | Spinach beverage | Beverage containing 800 g nitrate | Acute single doses crossover intervention (4 beverages) | 18 | Chemiluminescence technique, | Plasma (up to 300 min) | **nitrate**  **nitrite** | (111) |
|  | Spinach | Spinach containing 400 mg calcium | Acute single dose 6-arm cross-over intervention (5 food products incl. spinach and control) | 9 | Selective ion analyzer  Routine laboratory method | serum (over 7h) | **phosphate**  **magnesium** | (112) |
|  | Spinach cooked according to normal household practice–chopped spinach (test A) or stored after chopping (test B) | 308 g - 367 g spinach, | Acute single dose 4-arm cross-over trial (2x spinach meal, supplement, control) | 11 | HPLC method with fluorescence and DAD | serum (over 9h) | **5-CH_3_-H_4_folate**  **10-formyl-folic acid**  **Folic acid**  **10-HCO-H_2_folate**  10-formyltetrahydrofolate (oxidized to **10-HCO-H_2_folate or 10-formyl-folic acid)** | (113) |
|  | Thirteen spinach  cultigens tested and categorized as high- and Low-Lutein | 5 servings of 50 g spinach/week | 3-arm parallel intervention (12 weeks; high-L and low-L + control) | 10 | HPLC-PAD | serum | **Lutein** | (114) |
|  | 3 different protein components with added spinach and vegetable juice | Vegetable juice: 100 ml (containing tomato, carrot, celery, beet, parsley, lettuce, watercress, spinach and ascorbic and citric acids)  100 g canned spinach  + 125 g protein source (fish, beef or bacon) | Acute single dose 3-arm parallel intervention trial | 21 | GC- MS | whole blood, urine, gastric content (up to 4 hours) | N-Nitrosamine (N-nitrosodimethylamine) (NS) | (115) |
|  | Spinach-containing diet – frozen all leaf spinach thawed and cooked | 453 g/d spinach | 2-arm cross-over intervention (2x 3-weeks) | 7 | Atomic absorption spectrophotometry (Fecal, urinary, and dietary calcium and zinc levels)  kits for oxalate and hydroxyproline-assays | urine  faeces | **calcium**  **oxalate**  **hydroxyproline**  **calcium**  **oxalate**  **zinc** | (55) |
|  | Intrinsically labelled spinach | 200 g spinach | Acute single dose 2-arm parallel trial | 4 | Stable isotope method  LC-atmospheric pressure chemical ionization (APCI)-MS | serum (over 34 days) | **lutein** | (116) |
|  | Thawed spinach | Spinach containing 220 mg of nitrate | Acute single dose 2-arm cross-over trial | 26 | GC-MS | saliva (up to 210 min) | **nitrate**  **nitrite** | (6) |
|  | Processed or raw spinach | Spinach containing 9277 μg β-carotene/d | 2-arm cross-over intervention (2x 4 weeks carrot or spinach) | 8 | HPLC | plasma | **carotenoids (β-carotene)** | (117)  Only abstract available. |
|  | Vegetable together with fried rice | 200 g | 1^st^ study: Acute single doses (spinach, celery, onion, no vegetables)  2^nd^/3^rd^ studies confirmation: singles doses or combinations | 10 (and 3 for 2^nd^ and 3^rd^) | LC−MS (UPLC and Q-TOF MS) (untargeted metabolomics) | urine (up to 7h) | **4-guanidino-butanoic acid** (with an isoprene modification) | (54) |
|  | Spinach as part as a “carotenoid diet” carrot puree and chopped spinach | 165 g/d carrot and 250 g/d spinach | 4-arm cross-over intervention (4 diets incl. carotenoid diet x 9 days) | 19 | HPLC-absorbance detector | plasma | **α-carotene**  **β-carotene**  **lutein/zeaxanthin** | (118) |
|  | Boiled spinach with fat-rich meal | 75g spinach | Acute single doses 3-arm parallel intervention trial | 24 | HPLC-fluorometric detector | serum (up to 240 min) | **α -tocopherol** | (119) |
|  | Reconstituted  drink from  concentrated  freeze-dried  extract | 500 μg total folate | Acute single doses 4-arm cross-over intervention (days) | 13 | Microbiologic assay after thermal extraction and enzyme treatment | plasma | **folate** | (120) |
|  | Daily Spinach intake assessed with weighed records | 18.9 (31) g/d in the high serum folate group compared to 8.4 (11.3) in the low (<9 ng/ml serum folate) | Cross-sectional study | 70 | Chemiluminescent immunoassay  HPLC and enzyme immunoassay (ferritin and homocysteine) | serum and red blood cells  plasma | **folate**  **homocysteine** | (121) |
|  | Frozen spinach cooked | 500 g heated spinach (1185 nmol sum of folates) | Acute single doses 4-arm cross-over intervention (4 treatments incl. spinach) | 24 | Stable isotope dilution assay | plasma (up to 12 hours) and urine (up to 24) | **5-CH_3_-H_4_folate** | (122) |
|  | Homogenised spinach | 225 g/d | Single-arm intervention (16 days) | 8 | HPLC-UV  Immunoassays (B12, folic acid and homocysteine)  Tryazin assay (iron) | serum | **folic acid**  **homocysteine** | (123) |
|  | Spinach powder preparation | 10 g/d spinach (11.3 mg lutein and 3.1 mg β-carotene) | 3-arm crossover  Intervention trial (3x 3weeks spinach tomato or carrot juice or spinach) | 23 | HPLC-PAD-UV-vis | plasma | **β-carotene**  **β-carotene-5,6,6’,6’-diepoxide**  **lutein**  **phytoene**  **phytofluene**  **α-carotene**  **β-cryptoxanthin**  **α-cryptoxanthin**  **lycopene-16,16’-diol**  **carotenoid oxidation product** | (124) |
|  | Whole and homogenized spinach leaves | 294 g spinach | Acute single doses crossover intervention ( 5 interventions; strawberries, ascorbic acid, red wine, spinach and control) | 8 | HPLC-PAD | plasma (up to 24h) | **lutein**  **zeaxanthin**  **β-carotene** | (125) |
|  | Freeze-dried ^13^C labelled spinach | 5 g (providing 160 μmol methoxyflavonols, including 70 μmol TMM4’-glucuronide) | Acute single dose | 5 | UHPLC-MS^n^ | plasma (up to 24h) | **^13^C_17_TMM-glucuronide**  **^13^C_17_TMM-sulfate**  **^13^C_16_Patuletin-glucuronidesulfate-methyl**  **^12^C_1_^13^C_16_Spinacetinglucuronide-sulfate**  **^13^C_17_Spinacetinglucuronide-sulfate** | (57) |
|  | Thawed spinach and cooked in microwave | Meal A: 600 g spinach; Meal B: 300 g spinach; Meal C: 0.4 mg folic acid in water;  Meal D: folate-free control meal | Acute single doses 4-arm crossover intervention | 20 | Chemiluminescence assay | plasma (up to 10h) | folate | (126) |
|  | Spinach + oil | 150g spinach (9 mg lutein) | Acute single doses 3-arm crossover intervention (spinach; broccoli or control) | 8 | No information in abstract | plasma (up to 12 and then from 24 to 104h) | **lutein** | (127) |
|  | Chopped, frozen spinach heated for five minutes in a microwave oven + oil | 150 g/d spinach: 9 mg/d lutein, 4 mg/d β-carotene | 2-arm cross-over intervention (2x 21 days; tomato + spinach or spinach) | 9 | HPLC-UV-vis | plasma | **lutein**  **β-carotene** | (128) |
|  | Processed and pureed spinach and raw spinach | 113 g/d (~120 mL/d) pureed spinach and 39.0 g/d spinach * | 2-arm crossover intervention (2x4 weeks; carrot or spinach) | 8 | HPLC-UV-vis | plasma | **total β-carotene**  **all-*trans*-β-carotene**  t**otal-α-carotene** | (129) |
|  | Dried spinach powder | 10 g/d powder (providing  11.3 mg of lutein and 3.1 mg of β-carotene) | 4-arm cross-over intervention (4x2 weeks; carrot, tomato or spinach) | 23 | HPLC-MS | plasma | **all-*trans* retinoic acid** | (130) |
|  | Cooked spinach | 600 g spinach | Acute single doses crossover intervention (apple sauce, spinach or synthetic folic acid) | 1 | Stable isotope dilution assays based on LC–MS | plasma (up to 6 hour) | **5-methyl-H4folate** | (131) |
|  | Powder mixed with water | 6.2 g/d spinach powder (5 mg lutein/d) | 2-arm cross-over intervention (10 days; spinach or perilla) | 12 | HPLC-UV  Enzymatic assays  Gas chromatography | plasma | **β-carotene**  **lutein**  α-carotene | (132) |
|  | Diets containing 25g or 5g of neutral detergent fibre with 450 mg/day of oxalic acid (by means of spinach) | 100 g served every other day | 2-arm crossover intervention (2x 6 weeks; both with spinach) | 12 | Colorimetric methods  Atomic absorption spectrometry | plasma | **inorganic phosphorus**  **calcium**  **magnesium** | (133) |
|  | Creamed cooked spinach | 400 g spinach containing 3.5 μmol K1 | Acute single doses 2-arm crossover intervention (spinach or natto) | 6 | HPLC-fluorometric detection | serum (up to 72h) | **vitamin K_1_** | (134) |
|  | Pureed spinach (heated in microwave) | 300 g, 20.8 μmol t-β-carotene equivalents) or carrot for men and puréed spinach only (300 g, 20.0 μmol t-β-carotene equivalents) for women | Acute single doses intervention (carrot or spinach for men and only spinach for women | 14 | LC-APCI- MS  GC–MS | serum | **retinol**  **t-β-carotene** | (135) |
|  | Cooked chopped spinach | 92 g/d (11.93 mg lutein + 7.96 mg β-carotene) | 3-arm cross-over intervention (3x3 weeks; tomato, spinach or mixed products) | 20 | HPLC-UV-vis | plasma | **lutein**  **β-carotene**  **β-cryptoxanthin**  **zeaxanthin** | (136) |
|  | Chopped spinach  fed intragastrical | 600 ml liquid test meal providing 10 mg lutein, 7.1 mg β-carotene, and 0.3 mg zeaxanthin. | Acute single doses  3-arm cross-over intervention (tomato, carrot or spinach meals) | 10 | HPLC-UV-vis | duodenal contents  chylomicrons | **lutein**  **β-carotene**  **lycopene**  **lutein**  **β-carotene** | (137) |
|  | Cooked spinach | 255 g spinach was given, containing 15 mg lutein and 5.8 mg β-carotene. | Acute single doses crossover (4 treatments | 12 | HPLC-absorbance detectors | Plasma (up to 10h) (TRL fraction) | **lutein**  **lycopene** | (138) |
|  | Spinach  Supplemented meal : whole leaf spinach or chopped spinach both heated in microwave | 300 g/d spinach containing 1·7 and 24·6 mg β-carotene, 3·8 and 26 mg lutein, 0·22 and 0·60 mg folate and 26 and 93 mg vitamin C | 4-arm crossover intervention (4 days) | 69 | HPLC-UV-vis | plasma | **lutein**  **folate**  **vitamin C** | (139) |
|  | Cooked spinach  (heated in microwave) | 300g | Acute single dose 4-arm crossover intervention (spinach or lettuce or beetroot + control) | 12 | High Performance Ion Chromatography -UV | plasma (up to 24h) | **nitrate** | (140) |
|  | Meals served with either spinach or pumpkin | Daily two portions of 82 g of spinach (containing 1.5 mg and 0.7 mg of β-carotene/portion) | Parallel 2-arm intervention (3 weeks; pumpkin or spinach) | 13 | HPLC/ APCI LC-MS | Serum and feces | **β-carotene**  **lutein** | (141) |
|  | Diet high in nitrate vegetables lettuce or spinach during two periods of four consecutive days | Not mentioned in abstract | Acute single doses intervention | 12 | GC-MS | saliva (after 1h)  urine (24h) | **nitrite**  **nitrate**  **nitrate**  **total nitrosamines**  **N-nitrosodimethylamine** | (142)  Only abstract available. |
| **Lettuce** | Mean lettuce intake | Food record | Observational study | 46 | No information in abstract | plasma (end of each diet record) | **vitamin K_1_ (phylloquinone)** | (40)  Only abstract available. |
|  | Fresh romaine | 200 g | Acute single doses crossover intervention | 11 | HPLC-fluorometric detector | serum (up to 9h) | **vitamin K_1_ (phylloquinone)** | (103) |
|  | Mean lettuce intake | FFQ + 48h-recall | Cross-sectional study design | 51 | No information on carotenoid measurement | plasma  adipose tissue | **β-carotene**  **β-carotene** | (143)  Only abstract available. |
|  | Fresh lettuce | 250 g | Acute single dose | 11 | HPLC-electrochemical detection | plasma (up to 6h) | **quercetin**  **ρ-coumaric acid**  **caffeic acid**  **β-carotene**  **vitamin C** | (60) |
|  | Diet high in nitrate vegetables lettuce or spinach during two periods of four consecutive days | Unknown | Acute single doses intervention | 12 | GC-MS | saliva (after 1h)  urine (24h) | **nitrite**  **nitrate**  **nitrate**  **total nitrosamines**  **N-nitrosodimethylamine** | (142) |
|  | Raw lettuce (sliced) | 300 g | Acute single dose 4-arm crossover intervention (spinach or lettuce or beetroot + control) | 12 | High Performance Ion Chromatography - UV | plasma (up to 24h) | **nitrate** | (140) |
|  | Mean lettuce intake | 3-day diet records | cross-sectional study | 49 | HPLC | serum | **zeaxanthin**  **β-cryptoxanthin** | (144) |
| **Rocket salad** | Rocket salad beverage | Beverage containing 800 g nitrate | Acute single doses crossover intervention (4 beverages) | 18 | Chemiluminescence technique | plasma (up to 300 min) | **nitrate**  **nitrite** | (111) |
| **Endive** | Bowl of thick endive soup (300 g) with a slice of white bread and a glass of water | 9 mg kaempferol from endive | Acute single dose | 8 | Positive and negative ion electrospray LC/MS | plasma and urine (up to 24h) | **kaempferol-3-glucoside**  **kaempferol-3-glucuronide** | (63) |

Abbreviations: DAD, diode array detector; FFQ, Food Frequency questionnaire; NMR, Proton nuclear magnetic resonance; GC, Gas Chromatography; GC-MS, Gas Chromatography - Mass spectrometry; GL, glycyrrhizin; HPLC, High-performance liquid chromatography; LC-MS Liquid chromatography- mass spectrometry; LPL, plasma lipoprotein subfraction; PDA, photodiode array detector; TRF, Triacylglycerol-rich lipoprotein fraction; UV-vis, ultraviolet-visible.
